# Forebrain medial septum sustains experimental neuropathic pain

**DOI:** 10.1038/s41598-018-30177-3

**Published:** 2018-08-08

**Authors:** Mohammed Zacky Ariffin, Khairunisa Mohamad Ibrahim, Andy Thiam-Huat Lee, Rui Zhi Lee, Shou Yu Poon, Hwai Kit Thong, Eugene Hern Choon Liu, Chian-Ming Low, Sanjay Khanna

**Affiliations:** 1 Department of Anaesthesia, Yong Loo Lin School of Medicine, National University of Singapore, Singapore, Singapore; 2 Department of Pharmacology, Yong Loo Lin School of Medicine, National University of Singapore, Singapore, Singapore; 3 Department of Physiology, Yong Loo Lin School of Medicine, National University of Singapore, Singapore, Singapore; 4 Neurobiology & Ageing Programme, Life Sciences Institute, National University of Singapore, Singapore, Singapore

## Abstract

The present study explored the role of the medial septal region (MS) in experimental neuropathic pain. For the first time, we found that the MS sustains nociceptive behaviors in rodent models of neuropathic pain, especially in the chronic constriction injury (CCI) model and the paclitaxel model of chemotherapy-induced neuropathic pain. For example, inactivation of the MS with intraseptal muscimol (2 μg/μl, 0.5 μl), a GABA mimetic, reversed peripheral hypersensitivity (PH) in the CCI model and induced place preference in a conditioned place preference task, a surrogate measure of spontaneous nociception. The effect of intraseptal muscimol on PH was comparable to that seen with microinjection of the local anesthetic, lidocaine, into rostral ventromedial medulla which is implicated in facilitating experimental chronic nociception. Cellular analysis in the CCI model showed that the MS region sustains nociceptive gain with CCI by facilitating basal nociceptive processing and the amplification of stimulus-evoked neural processing. Indeed, consistent with the idea that excitatory transmission through MS facilitates chronic experimental pain, intraseptal microinjection of antagonists acting at AMPA and NMDA glutamate receptors attenuated CCI-induced PH. We propose that the MS is a central monitor of bodily nociception which sustains molecular plasticity triggered by persistent noxious insult.

## Introduction

Pain, especially chronic pain is aversive and debilitating and is often comorbid with cognitive deficits such as memory impairment and depression. Interestingly, more and more it is recognized that the aversive-fearful state of chronic pain is strongly modulated, at least in part, by a network of limbic and cortical structures that are traditionally associated with reward, defensive behaviors, and learning and memory. Among these cortico-limbic structures implicated in nociception is the medial prefrontal cortex (mPFC)^1–8^. Furthermore, hippocampal synaptic transmission and changes in the local neuropil are implicated in acute nociception in the formalin model and peripheral hypersensitivity (PH) in animal models of neuropathic and arthritic pain^9–20^. Interestingly, the mPFC and particularly the hippocampus are anatomically interlinked with the forebrain medial septum (MS)^21–25^. The neurons in the MS are excited by peripheral application of noxious stimuli^26^. Moreover, we have reported that MS facilitates formalin-induced acute nociception and affective behaviors^27,28^. Based on the anatomical and functional linkages of MS with the forebrain, we hypothesized that the MS is involved in the mediation of the aversive state in animal models of long-lasting pain. To test the possibility we have now investigated the effect of inactivation of MS neurons with intraseptal microinjection of the GABA mimetic, muscimol, on nociceptive-aversive responses in two different models of long-lasting neuropathic nociception, especially the chronic constriction injury (CCI) model of neuropathic pain. We have compared the effect of inactivation of MS with inactivation of rostral ventromedial medulla (RVM) since this region is implicated in facilitating experimental long-lasting nociception^29^.

In parallel to effect on aversive behaviors, we have explored the effect of MS manipulation on cellular responses in the spinal cord and mPFC of the CCI animals. We reasoned that if MS is integrated with the pain network, then manipulating MS should affect nociceptive responses in these two regions in parallel with an effect on nociceptive behaviors. Notably, phosphorylated extracellular signal-regulated kinase (pERK) in mPFC is associated with stimuli-induced aversion, a measure of affect, in the CCI model in male rat^30^. While, the spinal expression of phosphorylated p38 (pp38) is associated with mediation of PH in the CCI model^31–37^. We analyzed the effect of MS inactivation on CCI-induced ‘basal’ and peripheral sensory stimulus-induced ‘evoked’ levels of these cellular markers in CCI animals. We reasoned that an effect of MS inactivation on the level of these markers, especially the basal level will strengthen the notion that MS sustains basal or ongoing nociceptive processing in the neuropathic model.

In addition, we have tested whether excitatory glutamatergic transmission in MS mediates nociception in the CCI model. Glutamate is a ubiquitous transmitter in CNS. Indeed, the MS region has a variety of glutamate receptors, including NMDA and calcium permeable AMPA receptors, receives glutamatergic efferent from a number of CNS regions, including hypothalamic regions that may convey nociceptive information to MS, and is home to intrinsic glutamatergic neurons^38–49^. In the present study, the role of septal glutamatergic mechanisms in PH was explored using intraseptal microinjection of pharmacological antagonists at AMPA and NMDA glutamate receptors.

## Results

### General

It should be noted that the experimenters performing behavioral tests were blinded to the drug treatments throughout the study. Furthermore, the animal was allowed to recover for at least 5 days from the last surgical manipulation before behavioral testing was carried out. Generally, each animal underwent both thermal and mechanical test for nociception, performed on alternate days. The periods of behavioral measurement were sub-divided into 3 phases, namely (a) Basal (b) Post-ligation (c) Post-microinjection.

In the CCI groups of animals, the Basal and Post-ligation measurements were made from thirteen days to one day before sciatic nerve ligation and day seven till day ten after ligation, respectively. The Post-microinjection measurements were made on days twelve to fourteen after ligation in CCI groups of animals. Day twelve coincided with the first intraseptal microinjection of the drug or vehicle followed by test for paw withdrawal threshold (PWT). The PWT was again measured on day thirteen. The same drug (or vehicle), as on day twelve, was microinjected on day fourteen which was followed by measurement of paw withdrawal latency (PWL). The solutions were prepared fresh before microinjection. The animals were sacrificed after the test for PWL for histological analysis (see ‘*Effect of intraseptal muscimol on spinal and supraspinal cellular responses in CCI animals*’, below).

The microinjection was performed 5–15 min before the start of the behavioral testing. The behavioral tests were performed over a period of 45 min. Here it is notable that we selected muscimol to inactivate MS so as to provide comparison with previous report using formalin test of acute, but persistent experimental pain^28^. Intraseptal muscimol produces a prolonged inactivation of neural activity in MS^28^. Lidocaine was preferred for microinjection into RVM so as to replicate published reports^50,51^ for a comparison with effect of intraseptal manipulation on PH. Bilateral microinjection of lidocaine is known to attenuate neuropathic PH, presumably by attenuating descending inputs from the RVM that facilitate nociception^50–52^. We have not tested the effect of intraseptal lidocaine on nociception. However, intraseptal microinjection of local anesthetics and muscimol have similar neural effects insofar that they attenuate septal neural activity marked by block of septo-hippocampal theta activation induced on intra-cerebral stimulation^28,44^.

### Effect of intra-cerebral microinjection on PH in the CCI model

#### Intraseptal muscimol and CCI

**Figure 1:**
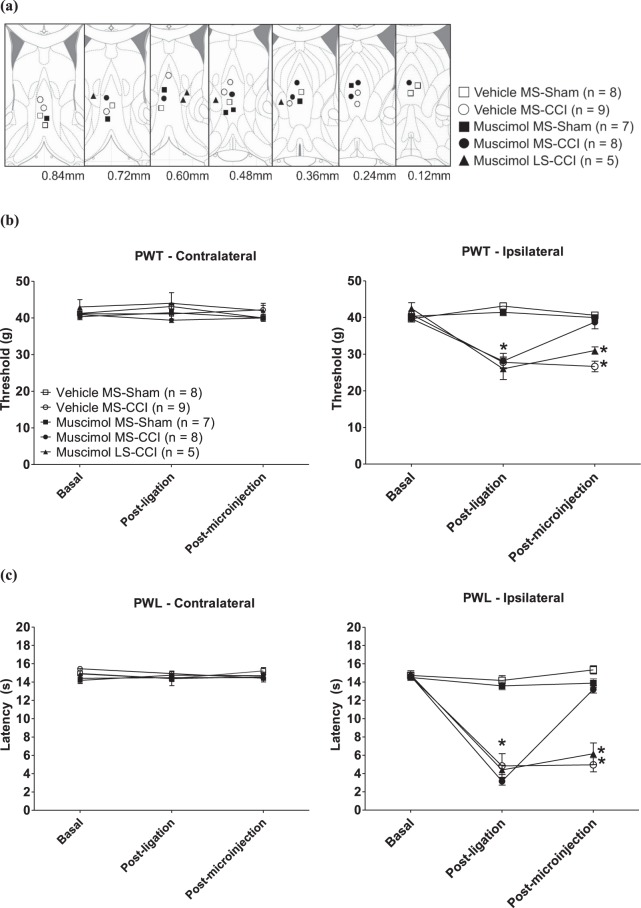
Muscimol in medial septum (MS) reversed peripheral hypersensitivity (PH) evoked on chronic constriction injury (CCI). (**a**) Diagrammatic representation of the microinjection sites. Muscimol (2 µg/µl, 0.5 µl) or the corresponding vehicle (0.9% sodium chloride, 0.5 µl) was microinjected into medial septum (MS), or the adjacent lateral septum (LS). The diagrams are adapted from Paxinos and Watson^95^. The value at the bottom of each diagram represents the stereotaxic coordinate anterior from the Bregma. The various groups illustrated follow the following nomenclature: drug (muscimol or vehicle), site (MS or LS) and peripheral manipulation (ligation or sham). (**b**) Paw withdrawal threshold (PWT) and (**c**) paw withdrawal latency (PWL) of the contralateral and ipsilateral hind paws in the CCI model following intra-septal muscimol microinjection. The ‘Basal’ and ‘Post-ligation’ measurements are averages of PWT and PWL made from thirteen days to one day before sciatic nerve ligation and day seven till day ten after ligation, respectively. The ‘Post-microinjection’ measurements of PWT were made on day twelve following the first intraseptal microinjection of the drug (or vehicle). The same drug (or vehicle), as on day twelve, was microinjected on day fourteen which was followed by measurement of PWL. Data points are mean ± S.E.M. Significant differences: *p < 0.05 vs. corresponding Basal value. Statistical analysis was performed using one-way repeated measures ANOVA. The line diagrams in the figure were taken from The Rat Brain in Sterotaxic Coordinates, 6^th^ ed., Paxinos G. and Watson C. Figures 26–32, Copyright Elsevier (2007).

In general, the PWT and PWL of the ipsilateral and contralateral paws were quite stable within a given phase. Thus, the data within the phase was combined to give a common value of PWT and PWL for the phase for each paw. Moreover, both PWT and PWL of the ipsilateral and contralateral paws were stable (Fig. 1b,c) across the different phases in Sham animals receiving intraseptal vehicle (0.5 μl; Vehicle MS-Sham: e.g. PWT ipsilateral paw, F_2,7_ = 0.89, p > 0.4, n = 8; PWL ipsilateral paw, F_2,7_ = 2.53, p > 0.1, n = 8) or muscimol (0.5 μl of 2 μg/μl muscimol solution; Muscimol MS-Sham: e.g. PWT ipsilateral paw, F_2,6_ = 1.22, p > 0.3, n = 7; PWL ipsilateral paw, F_2,6_ = 2.03, p > 0.1, n = 7). The lack of effect of muscimol in Sham animals suggests that MS is not crucial for physiological somatosensory reflexes.

On the other hand, muscimol microinjection into the MS reversed the PH induced on ligation of the right sciatic nerve. In this regard, repeated measure ANOVA followed by post-hoc comparison with Newman-Keuls test showed that ligation evoked a decrease in the PWT and PWL of the ipsilateral paw that was reversed to control on microinjection of muscimol into the MS (Fig. 1b,c; PWT: Muscimol MS-CCI: F_2,7_ = 31.79, p < 0.0001, n = 8; PWL: Muscimol MS-CCI: F_2,7_ = 374.90, p < 0.0001, n = 8). However, intraseptal vehicle did not affect the ligation-induced drop in the PWT and PWL of the ipsilateral paw (Fig. 1b,c; PWT: Vehicle MS-CCI: F_2,8_ = 86.18, p < 0.0001, n = 9; PWL: Vehicle MS-CCI: F_2,8_ = 46.15, p < 0.0001, n = 9). Microinjection of muscimol into the LS, which is contiguous with MS, evoked only a mild attenuation of the ligation-induced drop in ipsilateral PWT (Fig. 1b,c; Muscimol LS-CCI: F_2,4_ = 18.47, p < 0.002, n = 5) and PWL (Fig. 1b,c; Muscimol LS-CCI: F_2,4_ = 79.66, p < 0.0001, n = 5).

Ligation and subsequent microinjections of muscimol did not affect PWT and PWL on the contralateral side (Fig. 1b,c left panels; PWT: Muscimol MS-CCI: F_2,7_ = 0.89, p > 0.4, n = 8; PWL: Muscimol MS-CCI: F_2,7_ = 0.02, p > 0.9, n = 8).

#### Intra-RVM lidocaine and CCI

**Figure 2:**
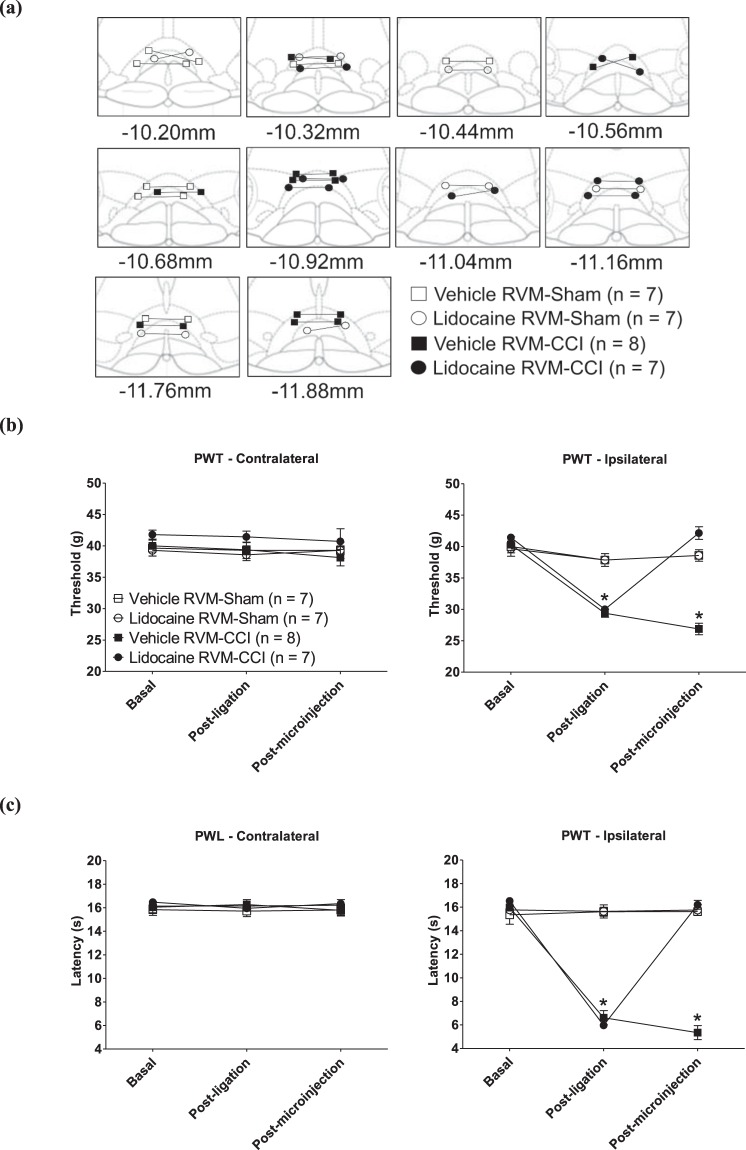
Muscimol in rostral ventromedial medulla (RVM) reversed peripheral hypersensitivity (PH) evoked on chronic constriction injury (CCI). (**a**) Diagrammatic representation of bilateral microinjection sites of the local anesthetic lidocaine (4% w/v, 0.5 µl) or the corresponding vehicle in Sham or CCI ligated animals. Diagram was adapted from Paxinos and Watson^95^ and built as in Fig. 1. The lines connecting the symbols indicate the pair of microinjection sites in each experiment. Bilateral microinjections of lidocaine, into the RVM was performed on days 12 and 14 after ligation or sham surgery that was followed by measurement of (**b**) PWT and (**c**) PWL, respectively. Data points are mean ± S.E.M. Notice that the y-axis scale in (**b**) ranges from 20–50 g while that in (**c**) the range is 4–20 s. This is different from Fig. 1 and other similar figures that follow Fig. 2. The scale was altered so as distinguish the line symbols more clearly. Significant differences: *p < 0.05 vs. corresponding Basal value. Statistical analyses were performed using one-way repeated measures ANOVA. The line diagrams in the figure were taken from The Rat Brain in Sterotaxic Coordinates, 6^th^ ed., Paxinos G. and Watson C. Figures 118–122; 124–126; 131–132, Copyright Elsevier (2007).

Bilateral microinjection of lidocaine (4% w/v, 0.5 µl/site) in ligated animals (Lidocaine RVM-CCI) also reversed PH on the ligated side. Repeated measure ANOVA followed by post-hoc comparison with Newman-Keuls test showed that PWT Post-ligation was significantly lower from the PWT during the Basal and Post-microinjection phases (Fig. 2b; Lidocaine RVM-CCI: F_2,6_ = 156.00, p < 0.0001, n = 7). The latter two were not different from each other in line with the anti-nociceptive effect of intra-RVM lidocaine. Similarly, PWL of ipsilateral (ligated) paw was reversed to control (Basal) following microinjection of lidocaine into RVM (Fig. 2c; Lidocaine RVM-CCI: F_2,6_ = 405.10, p < 0.0001, n = 7). On the other hand, microinjection of vehicle (0.5 µl/site) into the RVM of ligated animals did not affect the drop in ipsilateral PWT (Fig. 2b; Vehicle RVM-CCI: F_2,7_ = 106.60, p < 0.0001, n = 8) and PWL (Fig. 2c; Vehicle RVM-CCI: F_2,7_ = 159.70, p < 0.0001, n = 8) observed in these animals. Both vehicle and lidocaine did not affect the PWT (Vehicle RVM-CCI: F_2,7_ = 2.33, p > 0.10, n = 8; Lidocaine RVM-CCI: F_2,6_ = 0.30, p > 0.70, n = 7) and PWL (Vehicle RVM-CCI: F_2,7_ = 0.44, p > 0.60, n = 8; Lidocaine RVM-CCI: F_2,6_ = 1.93, p > 0.10, n = 7) elicited from the contralateral paw in ligated animals. Indeed, the PWT and PWL of the contralateral paw were unaffected by ligation, the values being stable across different phases of measurements.

In Sham animals, neither intra-RVM vehicle (Vehicle RVM-Sham: F_2,6_ = 0.07 at least, p > 0.90 at least, n = 7) nor intra-RVM lidocaine (Lidocaine RVM-Sham: F_2,6_ = 0.05 at least, p > 0.50 at least, n = 7) affected the reflexes elicited from the ipsilateral or the contralateral paw.

The lack of effect of lidocaine, microinjected bilaterally into RVM, on ‘physiological’ reflexes evoked by peripheral stimuli applied to the contralateral hind paw of the CCI animals and the hind paws in Sham animals is consistent with the published reports that show that RVM lidocaine spares physiological reflexes while preferentially blocking PH^50,51^.

### Effect of intra-cerebral microinjection on PH in the PAC model

In the PAC model, the Basal phase encompassed the 7 day period preceding the onset of the paclitaxel injection. The effect of PAC or its vehicle on PWT and PWL was tested on days 16 and 17 from the onset of PAC injection (i.e. Post-PAC). Intra-septal muscimol (0.5 μl of 2 μg/μl muscimol solution or 0.5 μl of its vehicle) was paired with thermal and mechanical stimulus on day 18 and 20, respectively, from the start of paclitaxel injections (i.e. Post-microinjection).

Paclitaxel treatment resulted in bilateral peripheral hypersensitivity, especially a drop in mechanical threshold which is consistent with reports in the literature^50,53,54^. As regards to thermal responses, the difference in the bilateral values in the Basal and Post-PAC periods was small and variable. Indeed, a variable or inconsistent effect of paclitaxel regimen is seen on response to thermal stimuli^54^. Thus, we did not focus on effect of microinjection on PWL in the paclitaxel model.

**Figure 3:**
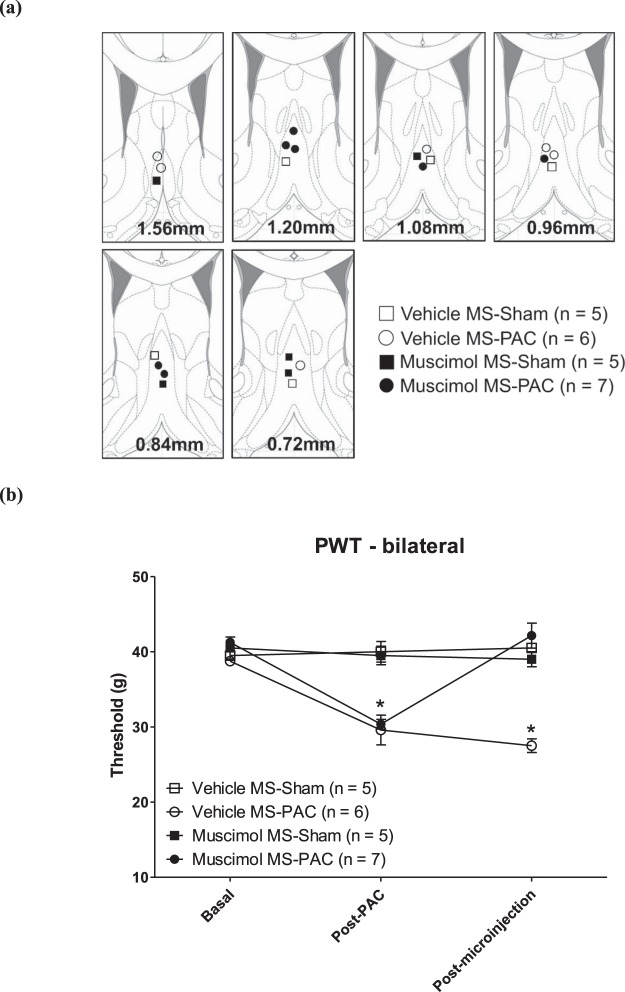
Muscimol in medial septum (MS) reversed mechanical allodynia evoked in the paclitaxel (PAC) model of neuropathic pain. (**a**) Diagrammatic representation of the microinjection sites. Muscimol (2 µg/µl, 0.5 µl) or the corresponding vehicle (0.9% sodium chloride, 0.5 µl) was microinjected into medial septum (MS). Diagram was adapted from Paxinos and Watson^95^ and built as in Fig. 1. The various groups illustrated follow the following nomenclature: drug (muscimol or vehicle), site (MS) and systemic treatment (PAC or Sham). (**b**) Bilateral paw withdrawal threshold (PWT) in the PAC model following intra-septal muscimol microinjection. PWT of the left and right paws were averaged to derive the bilateral PWT. The ‘Basal’ and ‘Post-PAC’ measurements are averages of PWT made from thirteen days to one day before start of PAC administration and day sixteen after the first PAC injection, respectively. The ‘Post-microinjection’ measurements of PWT were made on day eighteen following the first intraseptal microinjection of muscimol (or vehicle). Data points are mean ± S.E.M. Significant differences: *p < 0.05 vs. corresponding Basal value. Statistical analysis was performed using one-way repeated measures ANOVA. The line diagrams in the figure were taken from The Rat Brain in Sterotaxic Coordinates, 6^th^ ed., Paxinos G. and Watson C. Figures 20; 23–27, Copyright Elsevier (2007).

Muscimol microinjection into MS reversed the drop in PWT observed in this model (Fig. 3). Repeated measure ANOVA followed by post-hoc comparison with Newman-Keul’s test showed that PWT Post-PAC was significantly different from the PWT during the Basal and Post-microinjection phases (Fig. 3b; Muscimol MS-PAC: F_2,6_ = 49.40, p < 0.0001, n = 7). The latter two were not different from each other in line with the anti-nociceptive effect of intraseptal muscimol. On the other hand, microinjection of vehicle into the MS of paclitaxel treated animals did not affect the drop in bilateral PWT observed in these animals (Fig. 3b; Vehicle MS-PAC: F_2,5_ = 28.93, p < 0.0001, n = 6). In Sham animals, neither intra-MS vehicle (Vehicle MS-Sham: F_2,4_ = 0.83, p > 0.40, n = 5) nor intra-MS muscimol (Muscimol MS-Sham: F_2,4_ = 0.62, p > 0.50, n = 5) affected the reflexes elicited from the ipsilateral or the contralateral paw.

### Effect of intra-septal muscimol in conditioned place preference test in CCI animals

The CPP protocol was run from day 8 after ligation to day 11 after ligation. In the period preceding the CPP, the animals were tested for PH so as to verify that the ligation was effective in altering sensory responses. Microinjection (0.5 μl of 2 μg/μl muscimol solution) was performed about 5 min before introducing the animal to a compartment of the CPP apparatus on the test day (CPP3). The animals were sacrificed after the completion of the CPP protocol.

**Figure 4:**
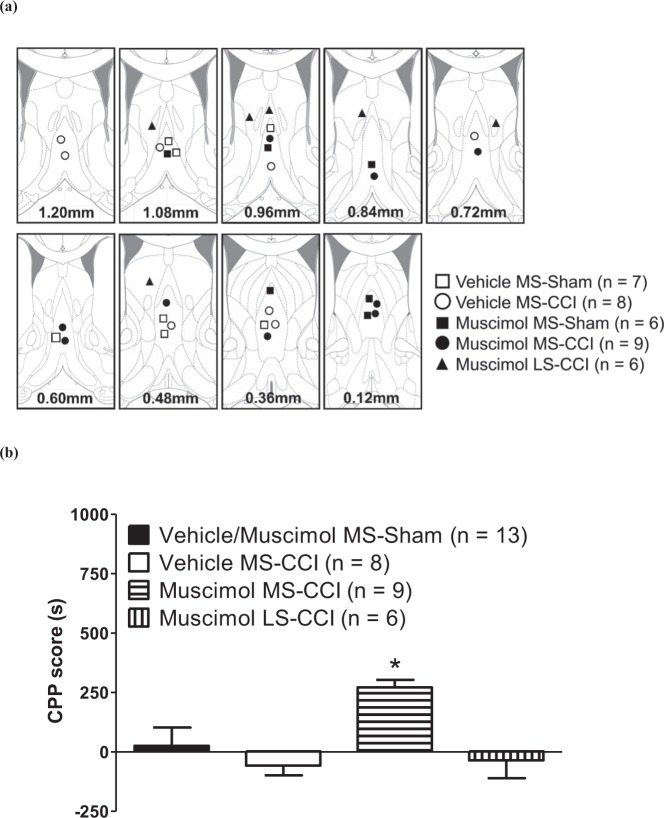
Muscimol, microinjected into medial septal region (MS), evoked conditioned place preference (CPP) in animals with CCI. (**a**) Diagrammatic representation of microinjection sites. Diagram was adapted from Paxinos and Watson^95^ and built as in Fig. 1. The various groups illustrated follow the following nomenclature: drug (muscimol or vehicle), site (MS or LS) and peripheral manipulation (ligation or sham). During conditioning day 3 or CPP3 (on 10^th^ day after ligation or sham surgery), the animals were microinjected with vehicle in the preferred compartment followed 3hrs later by another microinjection of either vehicle or muscimol which was, however, paired with the non-preferred chamber. The group nomenclature reflects the 2nd microinjection performed on CPP3. For the purpose of analysis, the Vehicle MS-Sham and Muscimol MS-Sham groups were combined (i.e. Vehicle/Muscimol MS-Sham) since they were not different from each other. (**b**) CCI animals’ microinjected with muscimol into MS exhibited conditioned place preference as indicated by the positive CPP score. CPP score was computed as the difference between the times spent in non-preferred compartment during CPP4 (i.e. on re-exposure after CPP3) and CPP2 (i.e. pre-conditioning session one day before CPP3). Data are mean ± S.E.M. Significant difference: *p < 0.05 vs. other experimental groups. Statistical analysis was performed using one-way ANOVA. The line diagrams in the figure were taken from The Rat Brain in Sterotaxic Coordinates, 6^th^ ed., Paxinos G. and Watson C. Figures 23–30; 32, Copyright Elsevier (2007).

During CPP3 of the conditioning protocol the animals were microinjected with vehicle (0.5 μl) in the preferred compartment followed 3hrs later by another microinjection of either vehicle or muscimol which was, however, paired with the non-preferred chamber. The CPP scores in Sham animals’ microinjected with either vehicle or muscimol into MS were similar. Therefore, these animals were grouped together to form the ‘Vehicle/Muscimol MS-Sham’ group (Fig. 4).

Interestingly, the microinjection of muscimol, though not the vehicle, as the 2^nd^ injection into MS altered animal place preferences in ligated animals without altering animal preferences in Sham animals (Fig. 4). However, muscimol microinjection into LS did not affect animal preference in ligated animals (Fig. 4). In context of the foregoing, analysis using one way ANOVA followed by Newman Keul’s test showed that CPP score for Muscimol MS-CCI animals was higher as compared to the other groups, the others being not different from each other (Fig. 4b; Groups, F_3,32_ = 4.89, p < 0.007).

In each of the experimental group shown in Fig. 4b there was no difference in average time spent in the preferred or the non-preferred compartment from CPP1 to CPP2 (p > 0.20 at least, two tailed paired t-test). Furthermore, there was no difference in the time spent in either the preferred or the non-preferred chamber on CPP1 and CPP2 among the different groups (Groups, F_3,32_ = 0.14 at least, p > 0.20 at least, one way ANOVA).

### Effect of intraseptal muscimol on spinal and supraspinal cellular responses in CCI animals

**Figure 5:**
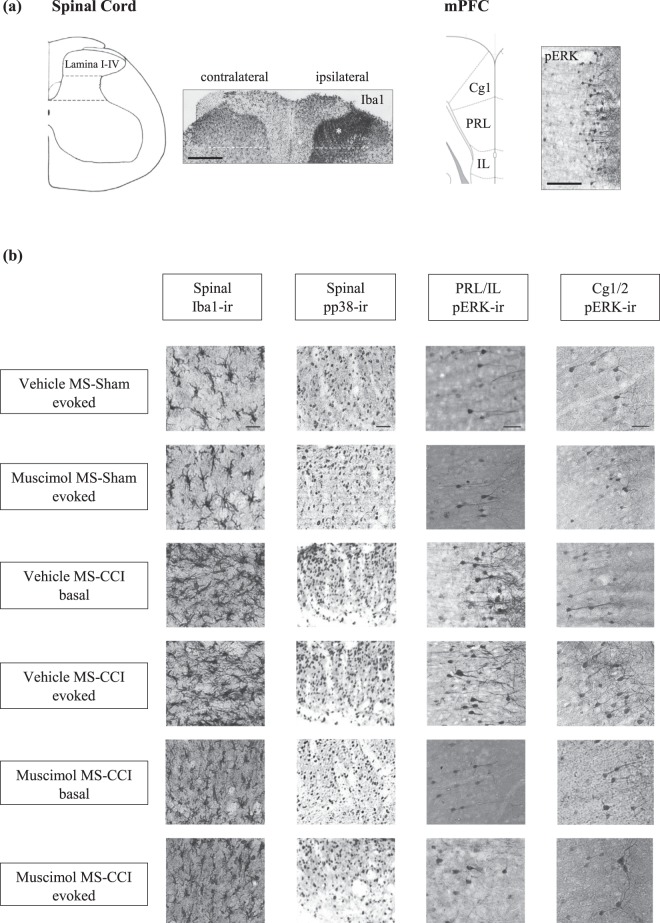
Muscimol, microinjected into medial septum (MS), modulates CCI-evoked cellular responses. (**a**) Diagrammatic representation of the lamina I-IV of lumbar spinal cord (left) and the mPFC (right). The diagrams are adapted from Paxinos and Watson^95^. The corresponding digital images shown on the right of the diagrams are of coronal sections taken through the spinal cord and mPFC from an experiment performed in the laboratory during the course of the present study. The inferior border of lamina IV of the dorsal horn is indicated by white dashed lines on the digital image. Notice the dense expression (in the region around*) of Iba1-like immunoreactivity (Iba1-ir; Iba1 on figure) in lamina I-IV of the ipsilateral spinal cord. Likewise, pERK-like immunoreactivity (p-ERK-ir; pERK on figure) is observed in the mPFC. Scale bars on spinal and mPFC images represent 200 µm and 100 µm, respectively. (**b**) Representative digital images at high magnification showing the pattern of cellular change across different experimental groups. Nomenclature of the groups is as described as in Fig. 1 with the addition of term ‘evoked’ or ‘basal’ at the end of the group name. The term ‘evoked’ indicates that the response was stimulus-evoked, i.e. data was obtained after the test for thermal hyperalgesia. In comparison, the ‘basal’ response is the baseline data in absence of any peripheral mechanical or thermal stimulation in CCI animals. The immunolabelled cells appear as darkly stained against a lighter background. Notice the relative high density of label in the spinal cord and PRL/IL regions of the ‘Vehicle MS-CCI basal’ and ‘Vehicle MS-CCI evoked’ groups as compared to other groups. Whereas, a relative high density of label is seen in Cg1/2 in the ‘Vehicle MS-CCI evoked’ group. Scale bars on the spinal and mPFC images in (**b**) represent 10 µm and 50 µm, respectively. The line diagrams in the figure were taken from The Rat Brain in Sterotaxic Coordinates, 6^th^ ed., Paxinos G. and Watson C. Figure 208d, Copyright Elsevier (2007).

**Figure 6:**
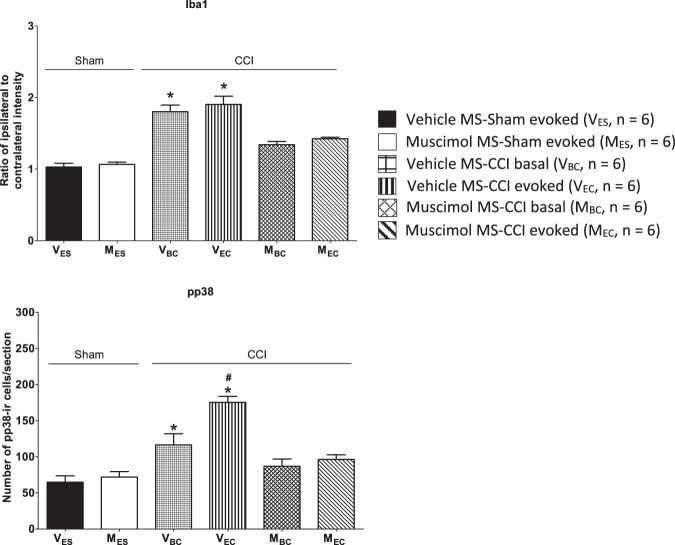
Muscimol, microinjected into medial septum (MS), attenuated CCI-evoked microglial activation in the spinal cord. Histograms of relative density of Iba1-ir expression (top; Iba1 on plot) and the number of neurons expressing phosphorylated p38-ir (pp38-ir; bottom; pp38 on plot) in lamina I-IV of the ipsilateral spinal cord dorsal horn. The nomenclature of the groups is as in Fig. 5. A ‘basal’ increase of Iba1-ir was observed in CCI model that was reduced on microinjection of muscimol into MS. On the other hand, the increase in pp38-ir was both ‘basal’ and ‘evoked’ both of which were attenuated on microinjection of muscimol into MS. Data are mean ± S.E.M. Significant difference: *p < 0.05 vs. other experimental groups, ^#^p < 0.05 vs. V_BC_ group. Statistical analyses were performed using one-way ANOVA.

**Figure 7:**
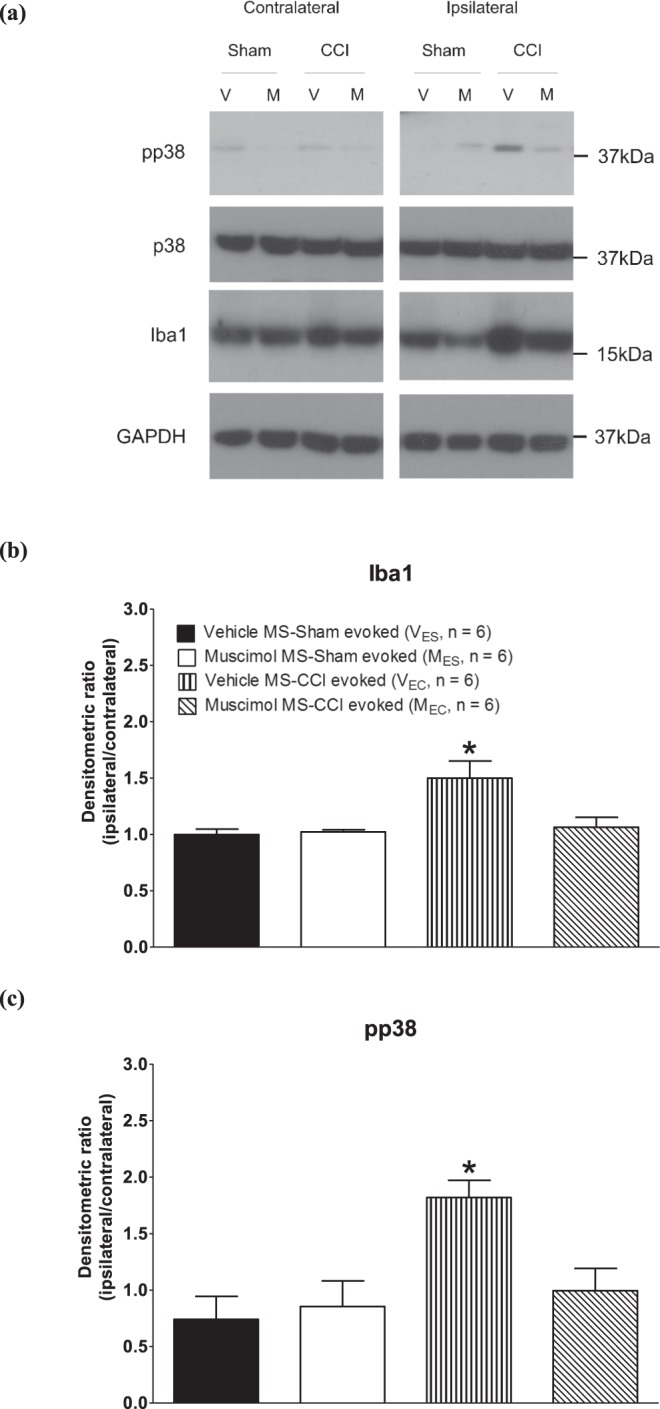
Western blot analyses of the effect of intraseptal muscimol on the level of microglial proteins in the spinal cord. (**a**) Representative immunoblots of the ipsilateral and contralateral lumbar spinal cord (20 µg of protein sample) from animals microinjected with vehicle (V) or muscimol (M) into the MS. Immunoblots were repeated for 6 animals in each experimental group. GAPDH was used as a loading control. Band images were cropped from full length blots which are presented in Supplementary Fig. 1. The nomenclature of the experimental groups is as in Fig. 5. Only tissue extracted from ‘evoked’ experiments were analyzed. (**b**,**c**) Densitometric ratio of the levels of proteins of interest. Individual band densities were first normalized to GAPDH band density of the same experimental group. Subsequently, the normalized values were used to calculate the ratio of the ipsilateral protein to the contralateral protein. Ratios were computed based on immunobands probed from the same membrane. Microinjection of muscimol in ligated animals decreased the protein level of Iba1 and pp38, which was otherwise high in Vehicle MS-CCI evoked group. Data are mean ± S.E.M. Significant difference: *p < 0.05 vs. other experimental groups. Statistical analyses were performed using one-way ANOVA.

**Figure 8:**
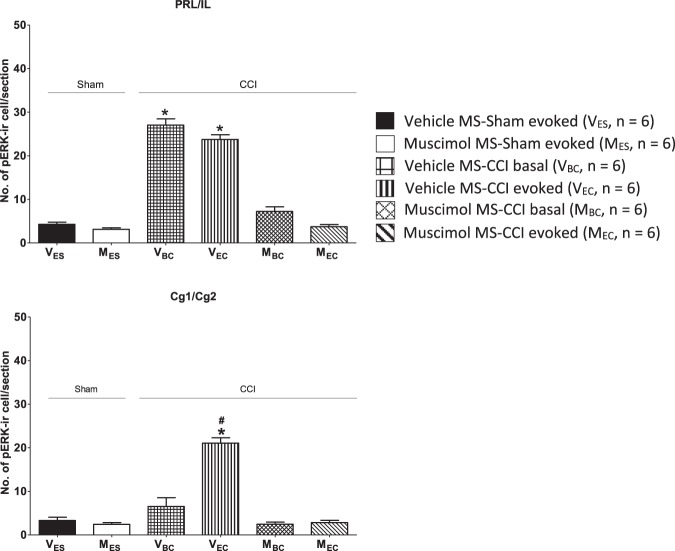
Muscimol, microinjected into the medial septum (MS), attenuated cellular index of nociception in medial prefrontal cortex (mPFC). Histograms of the number of neurons expressing pERK-ir in prelimbic/infralimbic (PRL/IL; top) and the cingulate cortical (Cg1/Cg2; bottom) regions of the mPFC. The nomenclature of the groups is as in Fig. 5. A ‘basal’ increase in pERK-ir was observed in the PRL/IL region of CCI animals that was reduced on microinjection of muscimol into MS. On the other hand, an intraseptal muscimol sensitive ‘evoked’ increase was observed in Cg1/Cg2 regions. Data are mean ± S.E.M. Significant differences: ^*^p < 0.05 vs. other groups, ^#^p < 0.05 vs. V_BC_ group. Statistical analyses were performed using one-way ANOVA.

Cellular analyses were performed on the spinal cord and the medial prefrontal cortex of the CCI groups of animals to determine the effect of intraseptal muscimol (0.5 μl of 2 μg/μl solution) on ‘basal’ and ‘evoked’ changes in cellular levels in the two regions (Figs 5–8). The ‘evoked’ cellular change refers to the level of cellular markers following application of peripheral heat stimulus in CCI animals. Four groups of animals comprised the ‘evoked’ group. These are the same group of animals as identified in ‘*Intraseptal muscimol and CCI*’ under ‘*Effect of intra-cerebral microinjection on PH in the CCI model*’. To identify the group as representing ‘evoked’ cellular change a suffix ‘evoked’ was added to group name.

In addition, two more groups of animals were included here. These are (i) Vehicle MS-CCI basal and (ii) Muscimol MS-CCI basal. These groups were used to gather matching ‘basal’ (baseline) data in absence of any peripheral thermal stimulation in CCI animals. The animals in the above two groups were ligated and handled in the same fashion as the other groups in the Basal and Post-ligation phases. However, they were left undisturbed in the experimental apparatus in the Post-microinjection phase after intraseptal microinjections of vehicle (0.5 μl; Vehicle MS-CCI basal) or muscimol (Muscimol MS-CCI basal) on day 12 and day 14 after ligation. To identify the group as representing ‘basal’ cellular change a suffix ‘basal’ was added to group name.

The animals were sacrificed an hour after drug (or vehicle) microinjection.

#### Spinal cord

The effect of intraseptal muscimol on spinal processing was analyzed using two cellular readouts of microglial activation, namely the expression of ionized calcium-binding adapter molecule 1-immunoreactivity (Iba1-ir) and the induction of pp38-ir. A dense aggregation of Iba1 positive microglia was observed in the dorsal region of the ipsilateral spinal cord, which corresponds to lamina I-IV, as compared to the contralateral side (Fig. 5a,b), which is consistent with the evidence in the literature^55^. We analyzed the change in ipsilateral Iba1-ir by analyzing the density of Iba1-ir in the ipsilateral spinal cord as compared to the density of the immunoreactivity in the contralateral spinal cord (relative density; Figs 5 and 6). Compared to Sham ligation, the relative density of Iba1-ir in dorsal area of the ipsilateral spinal cord was increased after ligation which, however, was reversed to levels observed in Sham groups on intraseptal microinjection of muscimol (Fig. 6; Groups, F_5,30_ = 28.14, p < 0.0001, one-way ANOVA followed by Newman Keuls’ test). The increase was observed in both the basal (Vehicle MS-CCI basal) and the evoked (Vehicle MS-CCI evoked) groups. The relative density of Iba1-ir in the two foregoing groups, however, was not different from each other.

As compared to Sham ligated animals, a significant increase in numbers of pp38-ir cells were also detected in the dorsal horn of the ipsilateral lumbar spinal cord of vehicle treated CCI animals which was reversed on intraseptal microinjection of muscimol (Fig. 6; Groups F_5,30_ = 14.92, p < 0.0001, one-way ANOVA followed by Newman Keul’s test). An increase was observed in both the basal (Vehicle MS-CCI basal) and the evoked (Vehicle MS-CCI evoked) groups of ligated animals. Interestingly, the number of pp38-ir cells in the Vehicle MS-CCI evoked group was significantly higher than in the Vehicle MS-CCI basal group, thus suggesting that the thermal test for PH evoked an expression of pp38-ir over and above the CCI-induced basal increase in pp38-ir.

Western blot analysis was also used to evaluate the effect of intraseptal muscimol on Iba1 and pp38 expression in the CCI model (Fig. 7a). Tissues were analyzed only from ‘evoked’ experimental groups. The density of individual band was quantified and normalized to the density of the corresponding GAPDH band. Subsequently, the relative density, i.e. the ratio of ipsilateral to contralateral spinal value, was used for analyses. In line with the immunohistochemical analyses described above, microinjection of muscimol in ligated animals (Muscimol MS-CCI evoked) decreased the protein level of Iba1 (Fig. 7a,b; Groups, F_3,20_ = 6.65, p < 0.003, one-way ANOVA followed by Newman Keul’s test) and pp38 (Fig. 7a,c; Groups, F_3,20_ = 7.80, p < 0.002, one-way ANOVA followed by Newman Keul’s test) which was otherwise high in Vehicle MS-CCI evoked group. In context of pp38, it is notable that the level of p38 protein was unaffected by ligation or with muscimol microinjection (data not shown) suggesting that the phosphorylation state of pp38 was selectively affected in the experiments.

#### Medial prefrontal cortex

The medial prefrontal cortex is organized in a dorsal-ventral axis into cingulate cortex, the prelimbic cortex and infralimbic cortex. A similar pattern of induction of pERK-ir was observed in PRL and IL regions. On the other hand, the expression in Cg1 and Cg2, while similar, was different from PRL and IL. Therefore, the counts of number of pERK-ir neurons from PRL and IL were combined and presented separately from the Cg1 and Cg2. The latter two were also grouped together. In addition, the induction of pERK-ir in layers II/III and V/VI of mPFC was bilaterally symmetrical. Thus, the bilateral values across different layers are presented here.

The increase in the number of pERK-ir neurons in PRL/IL reflected a ‘basal’ increase in ligated animals, whereas an ‘evoked’ increase was observed in Cg1/Cg2 (Figs 5 and 8). The microinjection of muscimol into MS reversed the ‘basal’ increase in PRL/IL to the level observed in Sham groups while preventing the ‘evoked’ increases in Cg1/Cg2 (Fig. 8). In context of PRL/IL, the number of pERK-ir neurons were similar in Vehicle MS-CCI basal and Vehicle MS-CCI evoked groups and significantly higher as compared to the count from the other groups, namely: Vehicle MS-Sham evoked; Muscimol MS-Sham evoked; Muscimol MS-CCI basal; Muscimol MS-CCI evoked (Fig. 8; Groups, F_5,30_ = 152.10, p < 0.0001, one-way ANOVA followed by Newman Keul’s test). The counts of other groups were not different from each other. In Cg1/Cg2, the count for Vehicle MS-CCI evoked group was significantly different from the other groups, the other groups being similar to each other (Fig. 8; Groups, F_5,30_ = 69.61, p < 0.0001, one-way ANOVA followed by Newman Keul’s test).

### Effect of intraseptal glutamate receptor antagonists on PH in the CCI model

**Figure 9:**
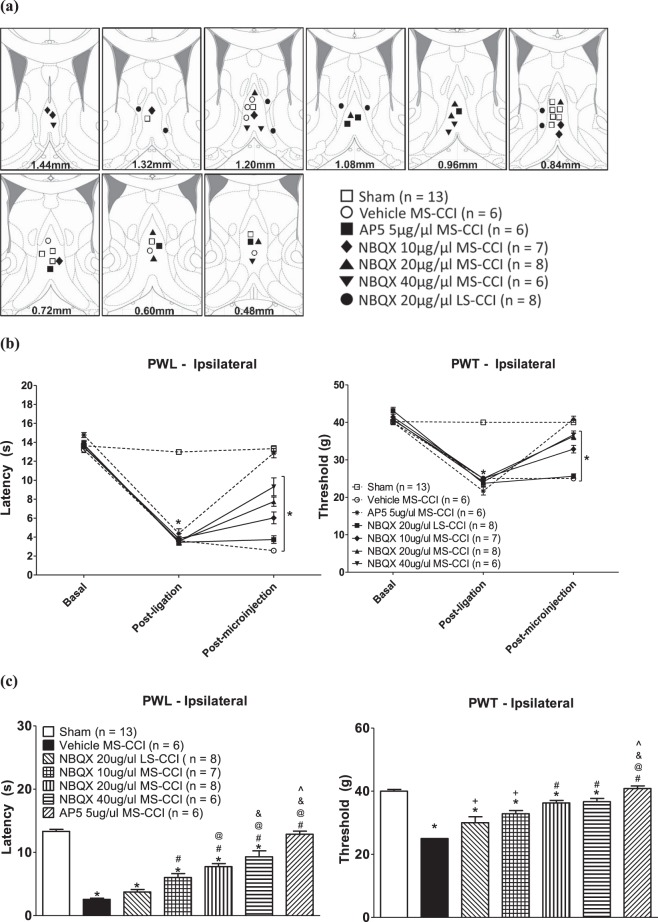
Septal glutamatergic neurotransmission contributes to CCI-induced peripheral hypersensitivity (PH). (**a**) Diagrammatic representation of microinjection sites. The diagrammatic representations are adapted from Paxinos and Watson^95^ and built as in Fig. 1. Varying concentrations of NBQX (10–40 µg/µl), an antagonist at AMPA glutamate receptors, or the selected concentration of AP5, an antagonist at NMDA glutamate receptors, was microinjected into the medial septum (MS). The various groups illustrated follow the following nomenclature: drug (NBQX, AP5 or vehicle) and its’ concentration, and site (MS or LS or MS and LS). Since the response of sham animals from the ‘Vehicle MS-Sham’ (n = 5) and the ‘NBQX 20 µg/µl MS-Sham’ (n = 8) were similar, they were grouped together and termed as ‘Sham’ group (n = 13). (**b**) Effect of ligation and drug microinjection on the paw withdrawal latency (PWL) and paw withdrawal threshold (PWT) of the ipsilateral hind paw. Line graphs are built as in Fig. 1. Note that the PWL and PWT of ‘Sham’ are stable across different periods of observations. Whereas, a sharp drop in PWL and PWT is seen ‘post-ligation’ in the remaining groups of CCI animals. A recovery towards Basal values of PWL and PWT is seen in ‘post-microinjection’ period in CCI animals following administration of NBQX and AP5. (**c**) Histograms depicting PWL (left) and PWT (right) observed in the ‘post-microinjection’ period in b. Note that the CCI-induced drop in PWL and PWT is attenuated on pre-treatment with NBQX and of AP5. Data are mean ± S.E.M. Significant differences: (**b**) *p < 0.005 vs. corresponding Basal value, one-way repeated measures ANOVA; (**c**) p < 0.05 as follows: * vs. ‘Sham’ group; +vs. ‘Vehicle MS-CCI’ group; # vs. ‘Vehicle MS-CCI’ and ‘NBQX 20 µg/µl LS-CCI’ groups; @ vs. ‘NBQX 10 µg/µl MS-CCI’ group; & vs. ‘NBQX 20 µg/µl MS-CCI’ group; ^ vs. ‘NBQX 40 µg/µl MS-CCI’ group. Statistical analyses were performed using one-way ANOVA in (**c**). The line diagrams in the figure were taken from The Rat Brain in Sterotaxic Coordinates, 6^th^ ed., Paxinos G. and Watson C. Figures 21–29, Copyright Elsevier (2007).

The three selected dose of NBQX (0.5 μl of either 10 μg/μl, 20 μg/μl or 40 μg/μl solution) and a dose of AP5 (0.5 μl of 5 μg/μl solution) were also examined for effect on PH in the CCI model (Fig. 9). The selected drug or vehicle (0.5 μl) was microinjected about 5 min before test for PH.

The control experimental groups consisted of sham ligated animals that received either Vehicle (Vehicle MS-Sham, n = 5) or 20 µg/µl of NBQX (NBQX 20 µg/µl MS-Sham, n = 8) in the MS. Neither sham surgery nor microinjection of vehicle or NBQX (20 μg/μl) into MS of these animals affected the PWL or PWT and the two groups were grouped together and termed as the ‘Sham’ group (Fig. 9). Indeed, the PWL and PWT of ‘Sham’ group were stable across different periods of observations (Fig. 9b; Sham: F_2,12_ > 0.37 at least, p > 0.20 at least, n = 13).

On the other hand, repeated measure ANOVA followed by post-hoc comparison with Newman-Keul’s test showed that ligation evoked a decrease in the PWL and PWT in the ‘post-ligation’ period, including in the Vehicle MS-CCI group (Fig. 9b). A trend towards recovery in the PWL and PWT was observed on microinjection of NBQX, but not vehicle into MS, though the PWL and PWT ‘post-microinjection’ were also significantly lower than the corresponding ‘Basal’ values (Fig. 9b). The statistics for various groups are as follows: Vehicle MS-CCI: F_2,5_ > 0.83 at least, p < 0.0001, n = 6; NBQX 10 μg/μl MS-CCI: F_2,6_ > 0.83 at least, p < 0.0001, n = 7; NBQX 20 μg/μl MS-CCI: F_2,7_ > 2.29 at least, p < 0.0001, n = 8; NBQX 40 μg/μl MS-CCI: F_2,5_ > 1.07 at least, p < 0.0001, n = 6; NBQX 20 μg/μl LS-CCI: F_2,7_ > 0.92 at least, p < 0.0001 at least, n = 8.

Whereas, microinjection of AP5 in ligated animals reversed the PH (Fig. 9b). In this context, repeated measure ANOVA followed Newman-Keul’s test showed that PWL Post-ligation was significantly lower from the PWL during the Basal and Post-microinjection phases (Fig. 9b; AP5 MS-CCI: F_2,5_ = 2.16, p < 0.0001, n = 6). The latter two were not different from each other in line with the anti-nociceptive effect of intra-MS AP5. Similarly, PWT of ipsilateral (ligated) paw was reversed to control (Basal) following microinjection of AP5 into MS (Fig. 9b; AP5 MS-CCI: F_2,5_ = 2.33, p < 0.0001, n = 6).

A comparison of Post-microinjection values for PWT and PWL among different groups using one way ANOVA followed by Newman-Keuls’ test (Fig. 9c; PWL: Groups, F_6,47_ = 75.73, p < 0.0001; PWT: Groups, F_6,47_ = 28.32, p < 0.0001) indicated that (a) PWT and PWL of Vehicle MS-CCI group was significantly lower than the corresponding values in ‘Sham’ animals, the ‘Sham’ being non-ligated control animals, (b) pre-treatment with microinjection of NBQX attenuated the drop in PWL and PWT, as compared to Vehicle MS-CCI group. The attenuation of PWL, but not PWT, was slightly but significantly greater at NBQX 40 μg/μl as compared to the two lower doses of NBQX, and (c) microinjection of AP5 attenuated both PWL and PWT such that the two were similar to the corresponding values in ‘Sham’ group and significantly higher that the PWL and PWT observed in Vehicle MS-CCI group.

Microinjection of NBQX (20 μg/μl) into LS also affected PH (Fig. 9c). However, the effect was relatively weak, being significantly lower than the effect seen on microinjection of the same dose into the MS (Fig. 9c). Finally, none of the manipulations affected PWT and PWL of the contralateral paw (data not shown).

## Discussion

The behavioral, cellular and pharmacological data in the present study converge to suggest that the MS is a nodal region wherein glutamatergic mechanisms mediate experimental neuropathic pain. Indeed, and in line with a facilitatory role of MS in nociception, inactivation of MS attenuated behavioral indices of nociception in the CCI model of long-lasting neuropathic pain. The MS region was inactivated with intraseptal microinjection of the GABA mimetic, muscimol, in a dose that is known to suppress septal neural activity^28,56–58^. Indeed, we and others have shown that intraseptal muscimol at the selected or lower doses attenuates hippocampal theta activation which is mediated by activation of septal neurons. Consistently, such muscimol-induced decrease in theta activation is accompanied by decreases in the utilization and release of acetylcholine in hippocampus and a decrease in theta related activity of neurons in the hippocampus. Here it is notable that the MS is the dominant source of cholinergic afferent to the region. The MS cholinergic neurons are implicated in nociception^38,59,60^. In the present study, muscimol microinjection into the MS, but not the adjacent LS reversed the mechanical and thermal PH after unilateral CCI injury of sciatic nerve. Likewise, inactivation of MS attenuated mechanical hypersensitivity in the paclitaxel model of chemotherapy induced neuropathic pain. The effect of intraseptal muscimol on PH was reversible and acute. In this context, while PH was attenuated for the duration for which the behavior was monitored (~45 min) post-microinjection, it was clearly observed the next day. Further, the effect of intraseptal muscimol on PH was comparable to bilateral microinjection of the local anesthetic, lidocaine, into the RVM, an area implicated in facilitation of experimental neuropathic pain.

Additionally, CCI animals exhibited a preference for the chamber in which they received an intraseptal microinjection of muscimol in the place-preference test. Intraseptal muscimol in sham animals had no such effect. This indirectly suggests that intraseptal muscimol also attenuated spontaneous nociception/aversion in the CCI model. The induction of preference for a chamber on septal inactivation is somewhat counter intuitive to the evidence that septal neural activity supports contextual learning^27,61,62^. Interestingly, however, the attenuation of nociception *per se* with intraseptal muscimol may drive animal preference in the CPP test via an interaction between the ventral tegmental nucleus and NAc which has been implicated in CPP in other models of pain^63–65^. The activation of such a system is suggested to reflect an activation of VTA-NAc dopamine neurons encoding motivational value that reflect a ‘reward’ due to antinociception (negative reinforcement)^63,66^. Strikingly, sciatic nerve injury *per se* is known to result in decrease in dopamine release in NAc to pharmacological agents that are associated with ‘reward’/addiction^25^.

In line with the behavioral data, inactivation of MS attenuated both basal and evoked cellular markers of nociception in the CCI model strengthening the finding that inactivation of MS was anti-nociceptive. In this context, a pattern of basal (pERK-ir in PRL and IL) or evoked (pERK-ir in Cg1/Cg2) expression of cellular markers was observed in the mPFC. It is notable that pERK in ACC is associated with stimuli-induced aversion, a measure of affect, in the CCI model in male rat^30^. Although the role of pERK in PRL/IL remains unclear, the region is implicated in neuropathic pain^67,20^. Additionally, the current findings show that spinal pp38-ir, which is associated with PH in CCI model (see introduction), exhibited both a basal and evoked increase in the CCI model. The phosphorylated state of a protein reflects a functional state of the protein during intracellular signaling and is generally dynamic. Consistently, the current findings using immunohistochemical and western immunoblot analysis show that while the spinal level pp38 levels are dynamic, varying with MS modulation, the level of the enzyme ERK in spinal cord is unaffected.

Collectively, the foregoing findings suggest a neural model wherein MS neural activity sustains nociception in the CCI model such that inactivation of MS dis-facilitates CNS basal nociceptive processing which attenuates cellular responses and spontaneous nociception. Further, the basal dis-facilitation prevents the amplification of neural processing on subsequent application of peripheral stimuli and blocks PH. Consistent with a role for septal neural activity in PH, intraseptal microinjection of the AMPA glutamate receptor antagonist, NBQX, or AP5, an antagonist at the NMDA glutamate receptor, attenuated PH. The foregoing suggests that septal glutamatergic transmission may underpin neural excitation in MS to sustain nociception in neuropathic models. Interestingly, an effect of NBQX, microinjected into the adjacent LS, was also observed on PH. However, the effect was weaker as compared to MS microinjections. Indeed, there was a functional separation between medial and lateral microinjections of drugs, whether muscimol or NBQX. The microinjections into MS evoked a robust attenuation of nociceptive indices while lateral microinjections into LS elicited little or no change in the indices. This strengthens the notion that key cellular elements mediating the effect of the microinjections in the present study are located more medially in MS. The weak effect seen on microinjection into LS might arise due to spread of drug into MS. Here it is also notable that the effect of the glutamate antagonists, and muscimol, on nociception is not linked to sedation since neither intraseptal muscimol nor the glutamate antagonists evoke a sedative like effect in rodent^28,68^. Neither have we observed any overt sedative-like impairment in animals in the present study.

Intriguingly, the current findings suggest that MS selectively facilitates an increase in nociceptive gain on the ligated side, while sparing physiological nociception. In context of the latter, the reversal of PH on the ligated side by intraseptal muscimol was marked by return of nociceptive thresholds towards normal. Further, microinjection of the GABA mimetic into the MS did not affect mechanical and thermal stimuli-induced reflexive withdrawal of hind limbs in sham animals or of the un-injured hind limb in CCI animals. Similarly, intraseptal NBQX or AP5 did not affect reflexive responses in the un-injured paw despite attenuating PH on the ligated side. The above distinction in the role of MS has a parallel in an electrophysiology finding which suggests that MS facilitates a ‘signal-to-noise’ processing in the hippocampal field CA1 to persistent (formalin) noxious stimulus^12^. The ‘signal-to-noise’ processing is marked by excitation of a population of putative pyramidal cells in CA1 against a background of MS mediated suppression of activity of majority of putative pyramidal cells. However, a ‘signal-to-noise’ response is not observed with brief noxious stimulus^12^. Moreover hippocampal theta wave activity, an electrophysiological measure of septal modulation of hippocampus, is long-lasting in the formalin test, while only a brief theta activation is observed with brief noxious stimulus^12,18^. Taken together, the preceding raises the possibility that MS modulation of nociceptive processing, and perforce the role septo-hippocampal network is heightened partly by the persistence of the noxious insult.

Interestingly, while the current findings suggest that inhibition of population of septal neurons with muscimol or attenuation of excitatory transmission through the region is antinociceptive, others reports indicate that high frequency electrical stimulation (HFS) of the septal region, presumably by exciting septal neurons and/or fibers in the region, is also antinociceptive in both anaesthetized and behaving animals^69–72^. The reasons for antinociception with divergent techniques are not clear. A possibility is that the different techniques affect distinct septal circuits. For example, HFS may trigger a circuit that evokes antinociception unlike intraseptal muscimol and glutamate antagonists that may impair a pro-nociceptive circuit in MS. Indeed, HFS, but not intraseptal muscimol/glutamate antagonists, at antinociceptive intensities can induce epileptiform after-discharges in the septo-hippocampal network and elicit behavioral changes such as wet dog shakes which suggests that electrical stimulation recruits a circuit with much wide-ranging sensory and motor effects^28,69,70,73^.

The nature of the input, and the pattern of cellular or molecular changes in the MS that heighten its role in neuropathic nociception is undefined. Current findings suggest that glutamatergic transmission, including glutamate transmission at the AP5 sensitive NMDA receptors may underpin MS mediation of neuropathic nociception, at least in part. Interestingly, MS receives glutamatergic input from thalamus and posterior hypothalamus, including supramammillary nucleus which is also associated with nociception^39,41,42,44–46^. Furthermore, the region receives spinal projections from NK-1 receptor positive neurons and glycinergic projections from RVM, especially the nucleus raphe magnus^74,75^ which is a component of the RVM. The pathway(s) via which MS facilitates persistent nociception also remains unclear. Indeed, MS is known to facilitate nociceptive processing in mPFC and hippocampus^12,27,76^. Interestingly, on one hand, the output from the hippocampus and the mPFC, especially PRL region converges, in part, on the NAc which has been proposed as a key regulator of both experimental and clinical chronic pain^7,77–83^. While, on the other hand, the output from mPFC also reaches the nucleus raphe magnus and spinal cord via PRL/IL and cingulate-spinal projection neurons, respectively^84,85^.

In summary, the present study brings out that the MS region is crucial to sustaining neuropathic nociception and, thus, a potential neural target for therapeutic manipulations to relieve chronic neuropathic pain. Indeed, the MS may function as a central monitor of bodily nociception that facilitates varied outcome to noxious stimuli of varied ranges. Thus, the septal neurons exhibit a diffuse and non-topographical receptive fields^26^ and, in turn, (a) modulate defensive behaviors, such as contextual fear, to brief noxious/aversive stimuli without affecting physiological reflexes evoked by the stimuli^27,86^,(b) facilitate ‘spontaneous’ nociception and avoidance behavior in the formalin test^27,28^. The ‘spontaneous’ nociceptive behaviors generally last for 1–2 hrs, and (c) sustain the long-lasting nociceptive gain and molecular plasticity triggered by persistent noxious insult (present study). Furthermore, septal neurons may participate in different circuits, at least in part, to modulate different behaviors. For example, in relation to (b) above, while MS facilitates both persistent nociceptive behavior and avoidance in the formalin model of persistent pain, avoidance, but not acute nociception is attenuated by destruction of GABAergic neurons in the MS^27,28^. Additionally, MS is implicated in anxiety^86,87^. Anxiety can be co-morbid with pain. Indeed, inhibition of septo-cingulate, but not septo-hippocampal cholinergic input attenuates the anxiety induced in animals that had previously experienced persistent experimental pain^60^.

## Methods

### Animals, implantation of cannula and generation of pain models

#### Animals

Experiments were performed on male Sprague-Dawley rats (300–350 g at the start of experiments) and followed the Ethical Guidelines of the International Association Study for Pain. All efforts were made to minimize the number of animals used. The experimental procedures were approved by the Institutional Animal Care and Use Committee (IACUC) at the National University of Singapore.

#### Implantation of microinjection cannula

All animals underwent survival surgery to implant a microinjection cannula in the brain. The rat was anaesthetized with isoflurane during surgical manipulations (5% isoflurane for induction and 2% for maintenance). A single stainless steel guide cannula (26 G, PlasticsOne, USA) was directed towards the MS (A0.5 mm, L0.0 mm from Bregma, V5.5 mm from skull surface) using stereotaxic techniques^27,28^. In animals where the RVM was targeted for microinjection, a double barrelled cannula with an inter-cannula distance of 1.2 mm (26 G, PlasticsOne, USA) was directed to the following coordinates: P2.5 mm from interaural line, L ± 0.6 mm from Bregma, V8.5 mm from skull surface^7^. Implants were secured to the skull with miniature screws and dental cement. A dummy cannula was inserted into the guide cannula to prevent occlusion of the lumen.

Post-implant, the animals were treated with an analgesic (Bupernorphine, 0.03 mg/kg, i.p.) and an antibiotic (Enrofloxacin, 10 mg/kg, i.p.) for three and five consecutive days, respectively.

#### Generation of pain models

A subset of the animals with implanted microinjection cannula into the septal region and the RVM were operated upon a second time at nine days after cannula implantation so as to ligate the right sciatic nerve. Ligation led to CCI of the nerve, a model of neuropathic pain. Other animals were injected with paclitaxel (PAC), a model of chemotherapy-induced neuropathic pain.

Ligation of the sciatic nerve: The right sciatic nerve was exposed following blunt dissection of the biceps femoris. The nerve was ligated by tying three chromic catgut sutures (4/0, Ethicon, Johnson & Johnson, India) around the nerve just proximal to the nerve trifurcation. The sutures were spaced 1 mm apart and were tightened to a point where the pressure elicited a slight twitch of the right hind paw. The muscle and skin layers were closed with nylon sutures (4/0, Supramid, Johnson & Johnson, India).

Sham ligated animals were surgically manipulated in the same way as the ligated animals except that the sciatic nerve was not ligated.

Peripheral injections: PAC (2 mg/kg; Anzatax, New Zealand) was administered intraperioneally once a day on alternate days across one week for a total of four injections. Thus, if day 1 was taken as the first day of PAC administration, the drug administration was repeated on days 3, 5 and 7. Such an administration of PAC leads to development of mechanical hypersensitivity^88^. Stock solution of PAC (6 mg/ml) was diluted with saline to a final concentration of 2 mg/ml which was used for administration. Animals in the PAC sham group were treated with vehicle injections of 3x dilution of 0.5 g/ml of Cremophor oil in ethanol.

### Behavioural procedures

#### Mechanical sensitivity

The paw withdrawal threshold (PWT) to mechanical stimulus was measured using an anesthesiometer (Ugo Basile, Italy). The animal was placed in a clear elevated chamber (L20 × W20 × H14cm) with a grid floor at least 10 minutes before the start of the experiment to facilitate habituation to the test chamber. Subsequently, mechanical pressure (20–50 g in 5 g increments, starting from 20 g) was applied to the plantar surface of the hind paws via a steel filament while the animal was alert, stationary, and on all four paws. A given pressure was applied six times to a paw. The stimulus was applied for a maximum duration of 6 s and was alternated between the two hind paws. The inter-stimulus interval between successive applications of mechanical stimulus to a given paw was at least 60 s.

#### Thermal sensitivity

Paw withdrawal latency (PWL) to thermal stimulation of the hind paw was tested using the plantar test apparatus (Ugo Basile, Italy). The animal was placed in an elevated chamber (L20 × W20 × H14cm) with a clear acrylic floor and allowed to habituate for at least 10 minutes before the onset of behavioural testing. A radiant heat source was placed under the chamber and directed towards the plantar surface of the hind paws while the animal was alert, stationary, and on all four paws. A thermal stimulus of 40 arbitrary units was applied alternately to the plantar surface of the two hind paws for a maximum duration of 20 s. The intensity of radiant heat was sufficient to evoke a paw withdrawal at around 15 s after the onset of the stimulus. The interval between successive applications of thermal stimuli to a given paw was at least 60 s. Application of thermal stimulus was repeated 5 times per hind paw.

#### Conditioned Place Preference (CPP)

The CPP apparatus consisted of two acrylic chambers (L46 x W46 x H40cm) that were separated by a black acrylic wall with a removable barrier (12 × 40 cm) positioned in the center of the wall. One chamber had a smooth floor with white colored walls (white chamber), while the other chamber had a pitted floor with black and white striped wall (black chamber). The entire apparatus sat in a cuboid frame with 20 infrared transmitters and receivers which were used to record and track animal behaviors and time spent in the individual chambers by Actimot program (TSE Systems, Germany). The experiments were performed in ambient light with the windows covered and the light switched off. The floors and walls were cleaned with 70% ethanol between uses.

The experimental procedure consisted of five sessions conducted over four consecutive days. These included one session each of preconditioning on days 1 and 2 (CPP1 and CPP2), two conditioning sessions on day 3 (CPP3) and a test session on day 4 (CPP4). During preconditioning, the barrier separating the chambers was removed, which allowed free access of both chambers to the animal. The animals were allowed to explore both compartments freely for 15 min in each session. At the beginning of each session, the animal was introduced into the middle of white chamber while facing the black wall. The time spent in each compartment was recorded automatically by ‘Actimot’ activity monitoring software (see above). The preference of the animal was noted and a biased protocol was used on day 3 (CPP3) whereby the animal was microinjected with muscimol in non-preferred compartment. Animals were excluded from the experiment if i) they showed extreme bias, that is they spent more than 80% of the time (720 s) in a single chamber during pre-conditioning, ii) switched preference on day 2, or iii) they showed more than 200 s difference in the time spent in the same compartment between day 1 and day 2. In the first session on conditioning day (CPP3), the animal was given an intraseptal microinjection of vehicle and confined to its preferred compartment for 1 hr. Three hour later, the same animal was given intraseptal microinjection of muscimol or vehicle and confined to the non-preferred compartment for 1 hr. On day 4 (CPP4), each rat was allowed to explore the two chambers freely for 15 min.

### Drug microinjection

The animal was gently restraint while a 33 G internal cannula (PlasticsOne, USA) was inserted into the implanted guide cannula. The internal cannula protrudes out from the tip of the guide cannula by 1.0 mm. The internal cannula was attached to an Exmire microsyringe (Ito Corporation, Japan) via polythene tubing. The drug was microinjected via the microsyringe over a period of 30 s. The internal cannula was left in place for 1 min after the microinjection to facilitate diffusion of the drug and to prevent backflow into the cannula track.

### Drugs

The following drugs were administered intracerebrally in the study: (a) muscimol hydrobromide (0.5 μl of 2 μg/μl solution; Sigma, USA), muscimol being a GABA mimetic^28^, (b) (2 R)-amino-5-phosphonopentanoate (AP5; 0.5 μl of 5 μg/μl solution; Sigma, USA) which is an antagonist at the glutamate NMDA receptors^68,89,90^, (c) 2,3-dihydroxy-6-nitro-7-sulfamoyl-benzo[f]quinoxaline-2,3-dione (NBQX; 0.5 μl of 10 μg/μl, 20 μg/μl or 40 μg/μl solution; Tocris, USA) which is an antagonist at glutamate AMPA receptor^39,92^, and (d) lidocaine hydrochloride (0.5 μl/site of 4% w/v; Sigma, USA), a local anesthetic^50^. The drugs were prepared in 0.5% Alcian blue dye in saline (Sigma, USA) which also acted as vehicle in control experiments. The drugs, (a) through (c), were used in experiments involving microinjection into the MS region. Intraseptal microinjections were made at one given site only, while, in comparison, lidocaine was microinjected bilaterally into the RVM.

Functionally, intraseptal microinjection of the fore-mentioned drugs at the selected concentration attenuates theta rhythmic septo- hippocampal activation^28,68,89–91^. The highest concentration of NBQX used in this study is also known to strongly attenuate afferent stimulation-evoked putative AMPA current in medial septal neurons^39^.

### Histology

Animals were deeply anaesthetized with urethane (1.5 g/kg, i.p.; Sigma, USA) immediately after the completion of test for thermal sensitivity on the last day of the experimental protocol for testing peripheral hypersensitivity (see below). The immunohistochemical labelling of brain and spinal tissue was performed as described previously^27,29,92,93^. Briefly, after perfusion and fixation with 4% paraformaldehyde (Sigma), the brain and/or spinal cord were sectioned into 60-μm coronal sections using a vibratome (Leica VT1200, Leica Microsystems GmbH, Germany). Serial sections were collected from the forebrain and incubated for 72hrs with rabbit anti-pERK antibody (1:1500; #4370, Cell Signaling, USA) to label neurons in mPFC. Two sets of alternate sections through L4 lumbar spinal cord were incubated with rabbit anti-Iba1 antibody (1:3000, #019–19741, Wako, Japan) and rabbit anti-pp38 antibody (1:500, #4511, Cell Signaling, USA). Subsequently, the sections were incubated overnight with secondary biotinylated goat anti-rabbit antibody (1:1000; Sigma, USA). The horseradish peroxidise-avidin-biotin complex (ABC, Vector Laboratories Inc., USA) was used to detect the antigen. Omission of the primary antibodies abolished the labeling.

Additionally, brain sections through the MS or RVM were mounted on gelatin coated slides, air dried, and stained with Cresyl violet (0.5% w/v; Sigma, USA) so as to identify the microinjection sites.

### Western blot

Immediately after the last behavioral test, the animal was decapitated and the lumbar spinal cord extracted. The tissue was washed in ice cold Tris-buffered saline (TBS) and dissected on ice to separate the left and right lumbar regions. The tissues were flash frozen in liquid nitrogen and homogenized with a mechanical douncer in 300 µl of denaturing radioimmune precipitation assay buffer (RIPA; 150 mM NaCl, 1% v/v Igebal, 0.5% w/v deoxycholic acid, 0.1% v/v SDS, 50 mM Tris, 1 mM EDTA, 1 mM EGTA) containing a cocktail of protease and phosphatase inhibitors (Complete and PhosphoStop; Roche, UK). The homogenate was centrifuged at 4 °C at 13200 rpm for 30 mins. The supernatant was retained and the protein concentration determined by Pierce BCA protein assay kit (Thermo Scientific, USA).

20 µg of protein was separated on 14% denaturing polyacrylamide gel (SDS-PAGE) and transferred to polyvinylidene difluoride (PVDF) membrane. Membranes were blocked in 5% w/v BSA in TBS-Tween (TBST) and probed with the following antibodies: mouse monoclonal anti-GAPDH (1:50000, MAB374, Millipore, USA), rabbit polyclonal anti-Iba1 (1:1000, #016–20001, Wako, Japan), rabbit monoclonal anti-pp38 (1:500, #4511, Cell Signaling, USA), and rabbit monoclonal anti-p38 (1:500, #9212, Cell Signaling, USA). After primary antibody incubation, the membranes were washed with TBST and incubated with horse radish peroxidase (HRP) conjugated goat anti-mouse antibody (1:50000, sc2031, Santa Cruz, USA) or goat anti-rabbit antibody (1:15000, sc2030, Santa Cruz, USA). Chemiluminesence was detected with ECL-Prime Western blot detection reagent (ECL Elite; Cell Signaling, USA).

### Data analysis

#### Quantification of nociceptive behaviors

Test of PH: The animal withdrawal of hind paw was signaled by flinching or lifting of the tested paw within 6 s and 20 s of the application of mechanical and thermal stimuli, respectively. The time of paw lift was automatically recorded by the anesthesiometer/plantar apparatus. The PWT for each paw was the pressure that elicited at least 4 positive responses (i.e. paw withdrawal). The PWL was measured as time taken in seconds to paw withdrawal. For each phase of the PH experiment, the PWT, and separately the PWL, were averaged for a given animal and then averaged across the group.

Test for CPP: The CPP score was computed as the difference between the time spent in the non-preferred chamber during the test session (CPP4) and the pre-conditioning session (CPP2). A positive score reflected a change in preference for the non-preferred chamber. The time spent in each chamber was monitored by using the Actimot program (TSE Systems, Germany). The CPP score was averaged across a given group.

#### Quantification of immunolabeling

The quantification of immunohistochemical label was performed as described previously^27,28,92,93^. Briefly, brain sections taken through mPFC, and spinal lumbar sections were digitized at 750dpi under 4x magnification (Nikon Eclipse E408 light microscope, Nikon Corporation, Japan) with the Montage Explorer software (Synoptics Ltd, UK). Immunolabel corresponding to pERK-, pp38-, and Iba1- immunoreactivity (ir) was analyzed with the MCID software (Interfocus Imaging Ltd, Niagara Inc, USA). The following criteria were adopted for analysis of IR: i) the intensity of immunolabeled neurons was at least 300% more intense than the background intensity, ii) the area of the label was >10 pixels for cytoplasmic label (pERK-ir) or >5 pixels for nuclear label (pp38- ir), and iii) form factor was ≥0.8 (form factor is a measure of degree of roundness, 1 indicates a perfectly round target). Iba1-ir was quantified by measuring the relative optical density (ROD) since a dense immunolabeling was detected which made it difficult to distinguish individual cells. The ROD was computed by the MCID analysis software by measuring the average grey level intensity per pixel. The number of pixels was determined by the size of the demarcated area i.e. dorsal horn spinal cord. The ROD thus reflects the ‘darkness’ of the region of interest in a black and white image. A larger ROD value indicates a higher intensity stain.

During immunohistochemical measurement, the background intensity was measured from a part of the stained section that was void of immunolabeling. Subsequently, the region of interest was outlined and the number of positively stained neurons that exceeded the background intensity was noted. pERK-ir in the mPFC (AP4.20 mm to −0.48 mm; 15–20 sections per animal) was analyzed bilaterally and separated according to sub-regions (prelimbic, infralimbic and cingulate regions) and lamina (lamina II/III vs. lamina V/VI) of the mPFC. Analysis of pp38- and Iba1- ir in the spinal cord dorsal horn region (lamina I to IV, 20–25sections per animal) was based on demarcation of the grey matter according to Molander *et al*.^94^.

The number of pERK-ir neurons counted on the contralateral and ipsilateral mPFC were averaged for the different lamina of the sub-regions for each section and subsequently across the total number of sections analyzed in each animal and then across each experimental group. Likewise for pp38- ir cells in the spinal cord. For Iba1-ir, ratio of the ipsilateral to contralateral ROD of the dorsal horn was computed and averaged across the number of sections analyzed in each animal and across each experimental group.

#### Densitometric analysis

The films were digitized and analyzed with the UN-SCAN-IT software. The density of each band was determined and normalized to the density of the GAPDH band in the same lane of the blot. The relative expression of protein on the ipsilateral side was calculated by computing the ratio between the GAPDH-normalized values on ipsilateral side divided by the corresponding value of the contralateral side.

#### Statistical analysis

Results are expressed as mean ± S.E.M and were analyzed using the Prism software (GraphPad Software Inc.). For a paired comparison of the changes in PWT and PWL across different phases of the experiment, the data were analyzed using repeated measure one-way ANOVA followed by Neuman-Keuls post-hoc test for multiple comparisons (Figs 1b,c, 2b,c and 9b). Comparison between multiple groups was performed with one way ANOVA, followed by Neuman-Keuls post-hoc test for multiple comparisons (Figs 3b, 4b, 6, 7b,c, 8, 9c). In some cases, a two-tailed unpaired t-test was used (see text). In instances, where Bartlett’s test showed unequal variance, the data were normalized by log or inverse transformation so as to equalize the variance and then analyzed. Statistical significance was accepted at least p ≤ 0.05.

The data analysed in the current study are available from the corresponding author on reasonable request.

## Electronic supplementary material

Supplementary Information 
