# Supplementary material for: Forebrain medial septum sustains experimental neuropathic pain
Source: Sci Rep. 2018 Aug 8;8:11892. doi: 10.1038/s41598-018-30177-3 (PMC6082830; doi:10.1038/s41598-018-30177-3)
Supplement: Supplementary file 1 — Supplementary Information [file 41598_2018_30177_MOESM1_ESM.pdf]

Forebrain medial septum sustains experimental neuropathic pain

Mohammed Zacky Ariffin[3, 4], Khairunisa Mohamad Ibrahim[3, 4], Andy Thiam-Huat Lee[3, 4], Rui Zhi Lee[3, 4], Shou Yu Poon[3, 4], Hwai Kit Thong[3,4], Eugene Hern Choon Liu[1], Chian-Ming Low[1, 2], Sanjay Khanna*[3, 4]

Departments of Anesthesia[1], Pharmacology[2], Physiology[3], Yong Loo Lin School of Medicine, and Neurobiology & Ageing Programme[4], Life Sciences Institute, National University of Singapore

* Corresponding author: phsks@nus.edu.sg, sanjay_khanna@nuhs.edu.sg

SUPPLEMENTARY INFORMATION

Supplementary Fig 1. Images of full length Western blots.

Full length blots for p38 and pp38

p38 (38kDa)

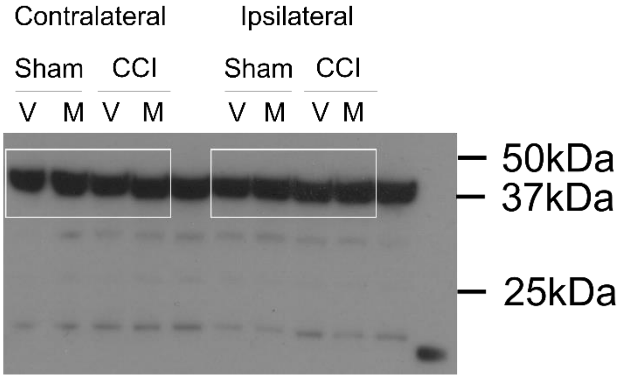

GAPDH (36kDa)

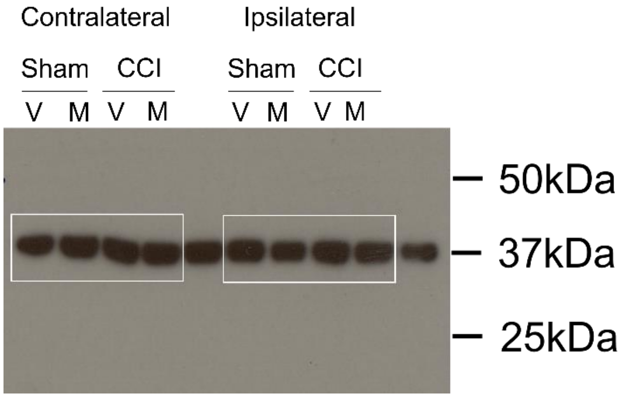

pp38 (38kDa)

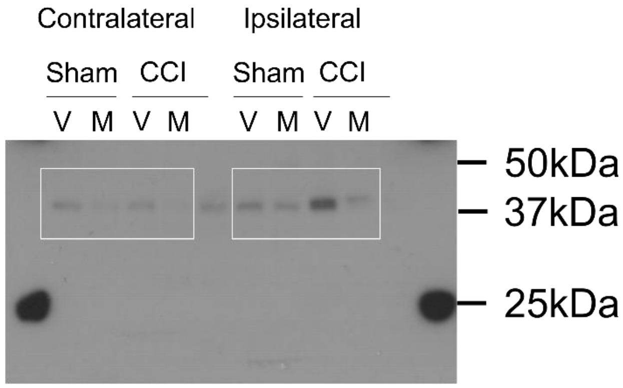

Full length blot for Iba1

Iba1 (17kDa)

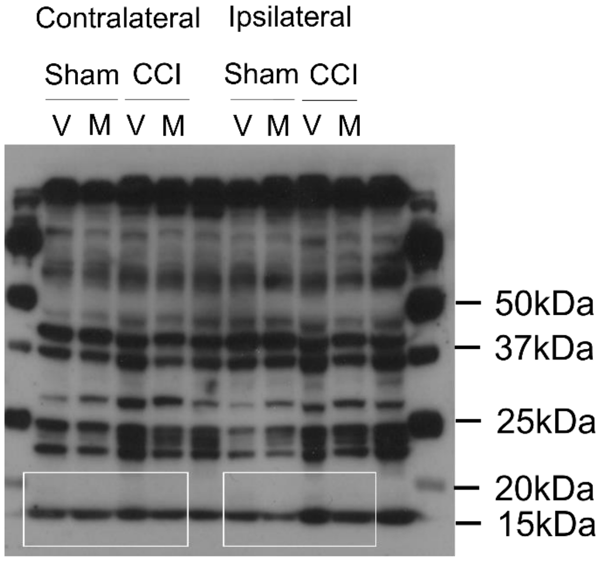

GAPDH (36kDa)

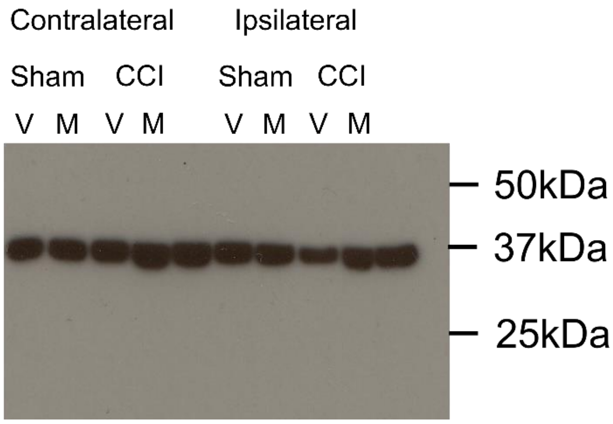

Supplementary Fig 1. Images of full length blots depicted in Fig 7. The blots for p38 and pp38 were probed from the same set of samples that was resolved on a single gel while the blot for Iba1 was derived from a separate set of samples. The corresponding blots for GAPDH are shown alongside. The blots for p38, pp38, and GAPDH are truncated as the PVDF membrane was cut prior to the blotting process. Scale on the right of each image was based on Bio-Rad Precision Plus Protein Standards molecular weight marker. The white boxes indicate the bands that are shown in Fig 7. Other bands observed in the blots were not used for analysis.
